# LncRNA GATA3‐AS1 facilitates tumour progression and immune escape in triple‐negative breast cancer through destabilization of GATA3 but stabilization of PD‐L1

**DOI:** 10.1111/cpr.12855

**Published:** 2020-07-20

**Authors:** Ming Zhang, Ning Wang, Peng Song, Yingqiang Fu, Yanlv Ren, Zhigao Li, Jinsong Wang

**Affiliations:** ^1^ Department of Breast Surgery Harbin Medical University Cancer Hospital Harbin China; ^2^ Department of Internal Neurology The First Hospital of Suihua City Suihua China; ^3^ Department of Orthopedics People's Hospital of Zhangqiu Jinan China

**Keywords:** COPS5, GATA3‐AS1, immune escape, PD‐L1, triple‐negative breast cancer

## Abstract

**Objectives:**

Long non‐coding RNAs (lncRNAs) have been demonstrated as crucial regulators in cancer, but whether they are involved in the immune response of cancer cells remains largely undiscovered. GATA3‐AS1 is a novel lncRNA that was upregulated in breast cancer (BC) according to online databases. However, its role in triple‐negative breast cancer (TNBC) was elusive.

**Methods:**

GATA3‐AS1 expression in BC tissues and adjacent normal tissues was obtained from online databases. Loss‐of‐function assays were designed and conducted to verify the functional role of GATA3‐AS1 in TNBC cells. Bioinformatic analysis and mechanism experiments were applied to explore the downstream molecular mechanism of GATA3‐AS1. Similarly, the upstream mechanism which led to the upregulation of GATA3‐AS1 in TNBC cells was also investigated.

**Results:**

GATA3‐AS1 was markedly overexpressed in TNBC tissues and cells. Knockdown of GATA3‐AS1 suppressed TNBC cell growth and enhanced the resistance of TNBC cells to immune response. GATA3‐AS1 induced the deubiquitination of PD‐L1 through miR‐676‐3p/COPS5 axis. GATA3‐AS1 destabilized GATA3 protein by promoting GATA3 ubiquitination.

**Conclusion:**

GATA3‐AS1 contributed to TNBC progression and immune evasion through stabilizing PD‐L1 protein and degrading GATA3 protein, offering a new target for the treatment of TNBC.

## 1 INTRODUCTION

Breast cancer (BC) is a kind of malignant tumour which threatens the health and happiness of women worldwide.^1^ Over the past decades, BC is characterized as high incidence and mortality in China.^2^ Histologically, BC can be divided into four major subtypes, including HER2‐enriched, luminal A, luminal B, and triple‐negative.^3^ Among all these subtypes, triple‐negative breast cancer (TNBC) is the most aggressive one, which is featured with higher recurrence and metastasis. So far, few targeted therapies were proven to be effective for TNBC. Therefore, exploring novel effective therapeutic targets is essential for the enrichment of therapeutic strategies.

Long non‐coding RNAs (lncRNAs) are characterized as the non‐protein coding long transcripts which is longer than 200 nucleotides.^4^, ^5^ Aberrantly expressed lncRNAs have been identified as the crucial biological participants in cancer progression.^6^ Thus, study on the biological role and molecular mechanism of lncRNAs in TNBC is valuable for finding novel therapeutic strategy in TNBC.

In our current study, GATA binding protein 3 antisense RNA 1 (GATA3‐AS1) was chosen as the research object in accordance with the online database. PD‐1/PD‐L1 checkpoint is acknowledged as the therapeutic scenario of advanced cancers in immunotherapy.^7^ However, some cases of human cancers failed due to the “immune escape”.^8^ In this study, we also examined the role of GATA3‐AS1 in immune escape.

Generally, cytoplasmic lncRNAs can regulate their downstream genes at post‐transcriptional level. In this study, the regulatory mechanism of GATA3‐AS1 on PD‐L1 protein was investigated. Since a previous report has indicated that the deubiquitination level of PD‐L1 protein was regulated by CSN5,22 this study explored whether GATA3‐AS1 regulated PD‐L1 through CSN5.

GATA binding protein 3 (GATA3), a nearby gene of GATA3‐AS1 was selected and detected. According to previous studies, GATA3 had prognostic significance in BC patients^10^ and acted as tumour suppressor in the progression of BC.^11^, ^12^ Recently, GATA3 has been reported as a regulator in the differentiation of T helper cells^13^ and the generation of functionally mature LTi cells.^14^ In this study, we explored the internal correlation between GATA3‐AS1 and GATA3 in TNBC cells. In summary, this study revealed the role of GATA3‐AS1 in regulating TNBC cell growth and metastasis and its downstream molecular mechanism.

## 2 MATERIALS AND METHODS

### 2.1 Clinical samples

The 68 matched samples of TNBC tissues and adjacent normal tissues were acquired between May 2014 and July 2019, with the approval of the Ethics Committee of Harbin Medical University Cancer Hospital. In our study, all patients provided the informed consent forms and none had received radiotherapy or chemotherapy prior to study. Tissue samples were snap‐frozen in liquid nitrogen and stored at −80°C after surgical resection for further analysis.

### 2.2 Cell culture and treatment

Human TNBC cell lines (MDA‐MB‐468, MDA‐MB‐436, MDA‐MB‐231, HCC1937) and human normal breast epithelial cell (MCF‐10A), from ATCC, were maintained in an incubator supplied with 5% CO_2_ at 37°C. DMEM medium (Thermo Fisher Scientific) was applied for cell culture with 10% FBS (HyClone, Logan, UT) and 1% Pen/Strep solution. Besides, MDA‐MB‐231 and HCC1937 cells were severally processed with C646 (20 μM) or DMSO (both, Sigma‐Aldrich). The protease inhibitor MG132 and cycloheximide (CHX) were all procured from Sigma‐Aldrich.

### 2.3 Total RNA extraction and quantitative real‐time PCR (qRT‐PCR)

According to the standard method, total cellular RNA was extracted using TRIzol method (Invitrogen) for cDNA synthesis using Power SYBR^®^ Green Master mix (Applied Biosystems). Gene expression was quantitated by qRT‐PCR with StepOne™ Real‐Time PCR System (Applied Biosystems), calculated with 2^−ΔΔCt^ method, relative to GAPDH or U6.

### 2.4 Plasmid transfection

The specific shRNAs and NC‐shRNAs were acquired from GenePharma Company for silencing GATA3‐AS1 and CBP in MDA‐MB‐231 and HCC1937 cells using Lipofectamine 2000 (Invitrogen). The pcDNA3.1/GATA3‐AS1, pcDNA3.1/GATA3, pcDNA3.1/COPS5 and NC vector, as well as miR‐676‐3p mimics/inhibitor and NC mimics/inhibitor, were all available from GenePharma Company. 48 hours later, cell samples were reaped.

### 2.5 Colony formation assay

Cell samples at logarithmic growth phase were placed in the 6‐well plates with 500 cells per well for 14‐day culture process. Samples were cultivated in crystal violet solution in 4% paraformaldehyde for counting.

### 2.6 EdU assay

Cell samples in 96‐well plates were prepared for EdU staining assay (RiboBio). After fixing and permeabilizing, cells were cultured with the DAPI solution and then assayed via fluorescence microscope (Olympus).

### 2.7 Transwell migration assay

Cells suspended in the serum‐free medium were planted in the upper chamber of transwell insert (Corning Incorporated), with the lower chamber supplemented with complete medium. Cells were dyed in 0.5% crystal violet after 24‐hours culture and then counted under microscope.

### 2.8 Chromatin immunoprecipitation (ChIP)

ChIP assay was implemented as instructed by the protocol of the EZ‐CHIP KIT (Millipore). Antibody against H3K27ac, CBP or control IgG was available from Millipore. Precipitated chromatin was assayed by SDS or qRT‐PCR.

### 2.9 Western blot

The extracted cellular protein samples were separated on the 12% SDS‐PAGE gel and shifted onto PVDF membranes and then treated with 5% skim milk. All of the specific primary antibodies and HRP conjugated to secondary antibodies were procured from Abcam and used after dilution. Samples were rinsed in TBST and then exposed to ECL Prime Western Blotting Detection reagent (GE Healthcare).

### 2.10 Co‐immunoprecipitation (Co‐IP)

Cellular lysates were acquired from the processed cells in IP lysis buffer and cultured with specific antibody at 4°C overnight in constant speed. Normal IgG served as negative control. After adding beads, the mixture was boiled in the SDS loading buffer for analysis.

### 2.11 Subcellular fractionation

Cytoplasmic or nuclear RNA was severally isolated form processed cells and purified employing PARIS™ Kit (Ambion) as per the user manual. GATA3‐AS1 expression level was monitored by qRT‐PCR.

### 2.12 FISH

The RNA FISH probe specifically designed for GATA3‐AS1 was available from RiboBio and utilized as required by supplier. Cell nuclei were all counterstained with DAPI for observing under microscope.

### 2.13 Luciferase reporter assay

MDA‐MB‐231 and HCC1937 cells in 48‐well plates were prepared for co‐transfection with 100 ng of pmirGLO luciferase reporter vectors (GATA3‐AS1‐WT/MUT or COPS5‐WT/MUT) and 50 nM of miR‐676‐3p mimics or NC mimics. 48 hours after transfection, Dual‐Luciferase Reporter Assay (Promega) was performed for luciferase activities. This assay was bio‐repeated for more than three times.

### 2.14 RNA pull down

RNA pull‐down assay was carried out as guided by the instruction of Pierce Magnetic RNA‐Protein Pull‐Down Kit (Thermo Fisher Scientific). Protein extracts from MDA‐MB‐231 and HCC1937 cells were processed with 50 pmol of biotinylated miR‐676‐3p probes and beads for 1 hour. The relative enrichment of GATA3‐AS1 or COPS5 was monitored by qRT‐PCR.

### 2.15 Flow cytometry

Transfected MDA‐MB‐231 and HCC1937 cell samples were co‐cultured with CD8^+^ T cells. Antibody against PD‐1 or PD‐L1 (Abcam) was added, and flow cytometry was then conducted for assessing the percentage of CD8^+^ T cells or CD8^+^ T‐cell apoptosis. This assay was bio‐repeated for more than three times.

### 2.16 Statistical analyses

Bio‐triple repeats were required for each experiment. The measurement data were exhibited as the mean ± standard deviation (SD) and analysed using PRISM 6 (GraphPad). Comparisons of groups were analysed via Student's *t* test or one‐way ANOVA *P* < .05 was considered statistically significant.

## 3 RESULTS

### 3.1 Overexpressed GATA3‐AS1 contributed to TBC cell proliferation and migration

**FIGURE 1:**
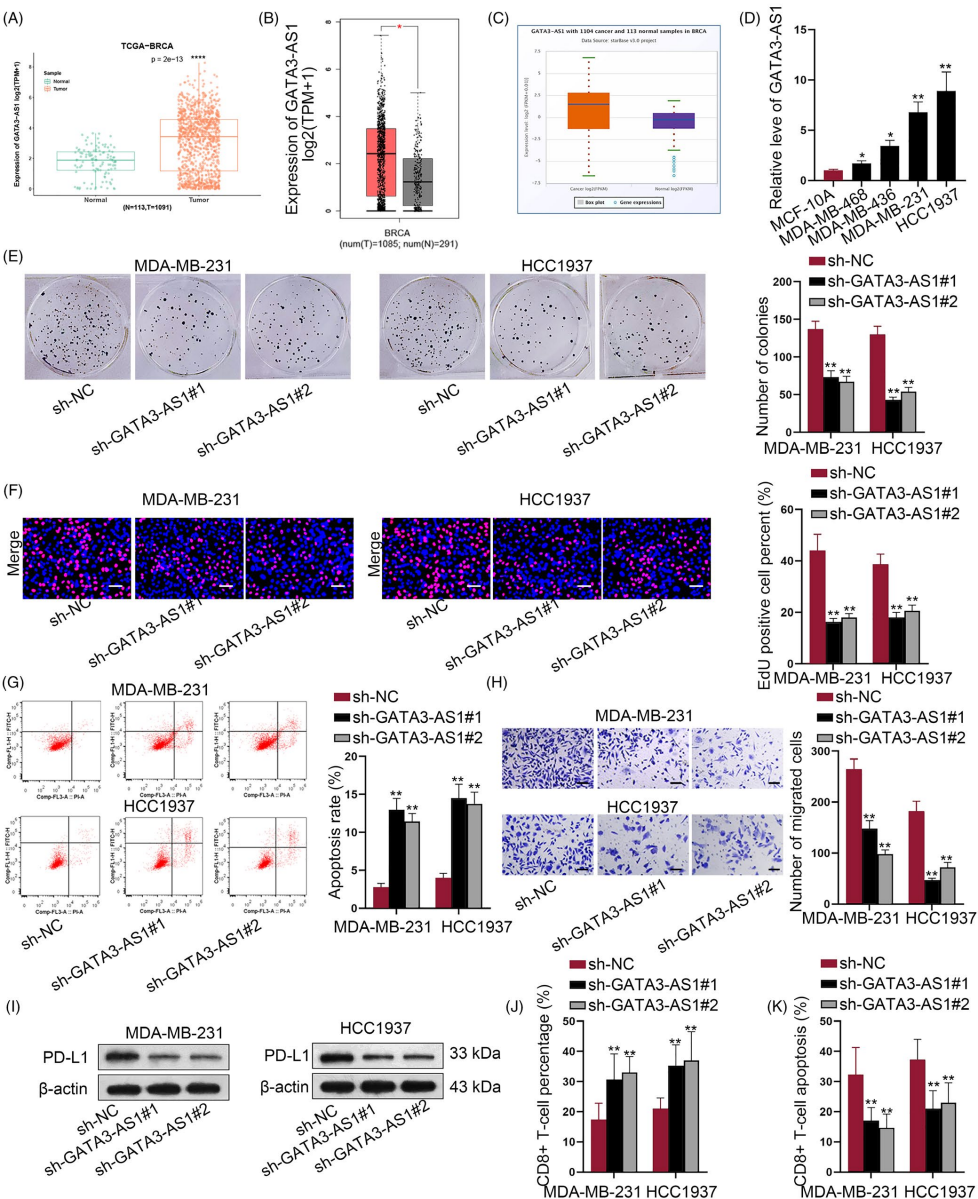
Overexpressed GATA3‐AS1 contributed to TNBC cell proliferation and migration. A‐C, High level of GATA3‐AS1 in BC samples was determined in accordance with three online databases, TCGA, GEPIA and starBase 3.0. D, The expression pattern of GATA3‐AS1 was determined in TNBC cells and normal MCF‐10A cell. E‐H, Proliferative ability, apoptosis rate and migratory ability of two transfected TNBC cells were assessed through colony formation assay, EdU assay, flow cytometry analysis and transwell migration assay, respectively. I, The protein level of PD‐L1 was assessed in TNBC cells transfected with GATA3‐AS1–specific shRNAs compared to sh‐NC–treated cells. J‐K, The percentage and the apoptosis rate of CD8^+^ T cells were observed under the silencing of GATA3‐AS1 in TNBC cells. *P < .05, **P < .01

Based on online databases, we determined that lncRNA GATA3‐AS1 was highly expressed in BC samples (Figure 1A‐C). Then, GATA3‐AS1 had high expression in four TNBC cells, especially MDA‐MB‐231 and HCC1937 cells (Figure 1D). However, GATA3‐AS1 had no aberrant expression in other subtypes of BC cells (Figure S1A). qRT‐PCR analysis showed that GATA3‐AS1 was efficiently knocked down by two specific shRNAs (Figure S1B). Functionally, cell proliferation was repressed by silencing GATA3‐AS1 (Figure 1E,F). However, apoptosis rate was enhanced in GATA3‐AS1–downregulated TNBC cells (Figure 1G). Likewise, silenced GATA3‐AS1 suppressed cell migration (Figure 1H). Intriguingly, GATA3‐AS1 silence reduced PD‐L1 protein level (Figure 1I) but did not affect PD‐L1 mRNA level (Figure S1C). Then, we determined that the percentage of CD8^+^ T cells was increased but the apoptosis rate of CD8^+^ T cells decreased after silencing GATA3‐AS1 in TNBC cells (Figure 1J,K). To obtain further evidence, we conducted gain‐of‐function assays in MDA‐MB‐468 and MDA‐MB‐436 cells. After GATA3‐AS1 was overexpressed in above two TNBC cells (Figure S1D), cell growth and migration were promoted (Figure S1E‐G). GATA3‐AS1 upregulation increased PD‐L1 protein level but did not affect PD‐L1 mRNA level (Figure S1H). Additionally, the decreased percentage of CD8^+^ T cells and decreased apoptosis rate of CD8^+^ T cells were observed in GATA3‐AS1–upregulated cells (Figure S1I,J). These findings implicated the oncogenic property of GATA3‐AS1 in TNBC.

### 3.2 GATA3‐AS1 prevented PD‐L1 protein against the ubiquitination in TNBC cells

**FIGURE 2:**
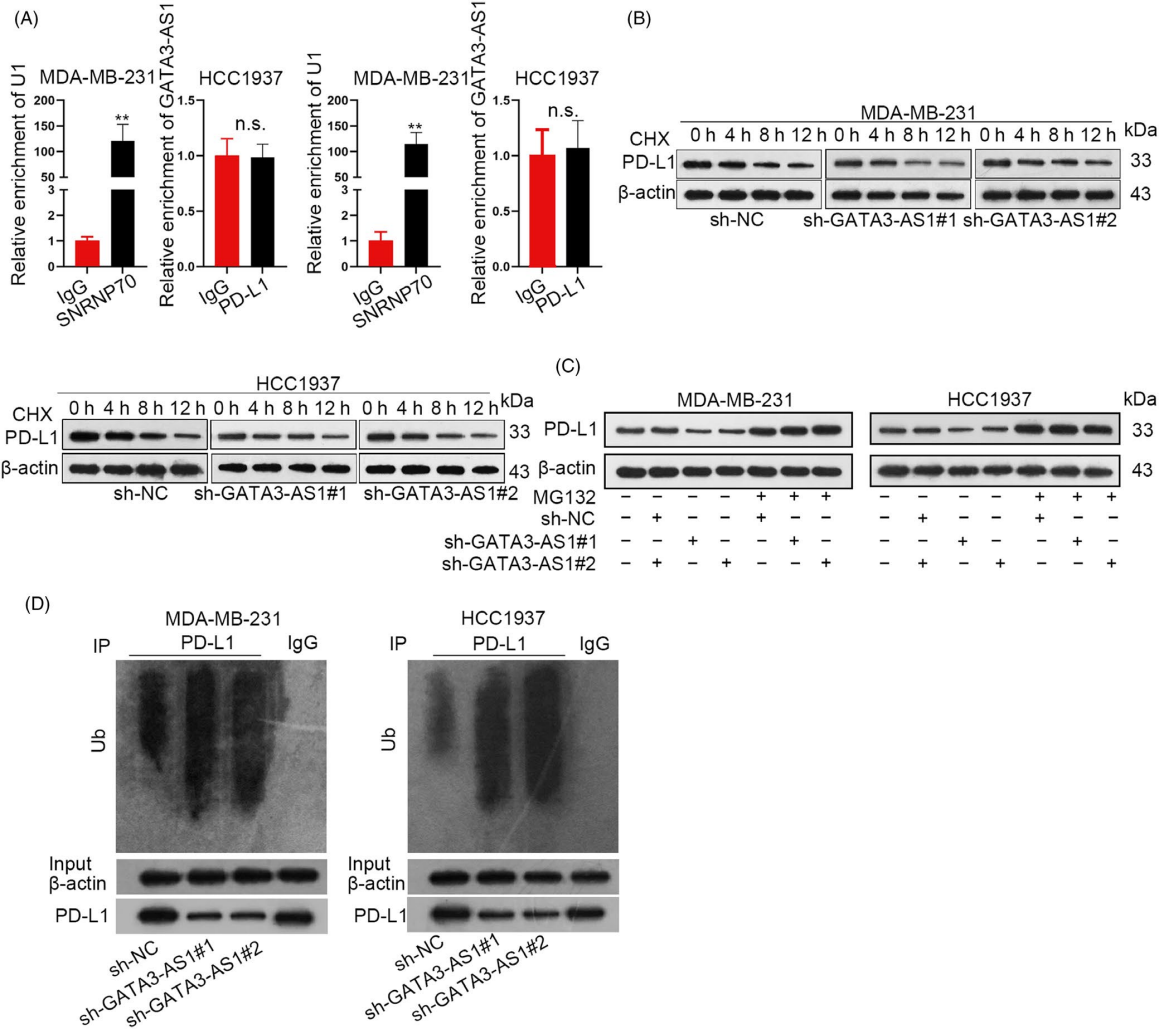
GATA3‐AS1 prevented PD‐L1 protein against the ubiquitination in TNBC cells. A, RIP assay was performed in TNBC cells to determine that whether GATA3‐AS1 could directly interact with PD‐L1. B‐C, Two TNBC cells separately incubated with MG132 (the proteasome inhibitor) and CHX (the protein synthesis inhibitor) were subjected to Western blot analysis after silencing of GATA3‐AS1. D, Ubiquitination assays were carried out in cells transfected with sh‐GATA3‐AS1#1/2 or sh‐NC to evaluate the ubiquitination level of PD‐L1. **P < .01. n.s.: not significant

According to RIP assay, we determined that GATA3‐AS1 could not directly interact with PD‐L1 in TNBC cells (Figure 2A). Then, Western blot analysis revealed that GATA3‐AS1 silence could decrease the half‐life of PD‐L1 protein (Figure 2B). As indicated in Figure 2C, PD‐L1 level was increased by MG‐132 treatment, while the increased level was unchanged by GATA3‐AS1 knockdown. Data from ubiquitination assays demonstrated that the ubiquitination level of PD‐L1 was obviously enhanced by GATA3‐AS1 depletion (Figure 2D). These data suggested that GATA3‐AS1 could stabilize PD‐L1 protein by inhibiting PD‐L1 ubiquitination.

### 3.3 GATA3‐AS1 positively regulated COPS5/CSN5 to reverse the deubiquitination of PD‐L1

**FIGURE 3:**
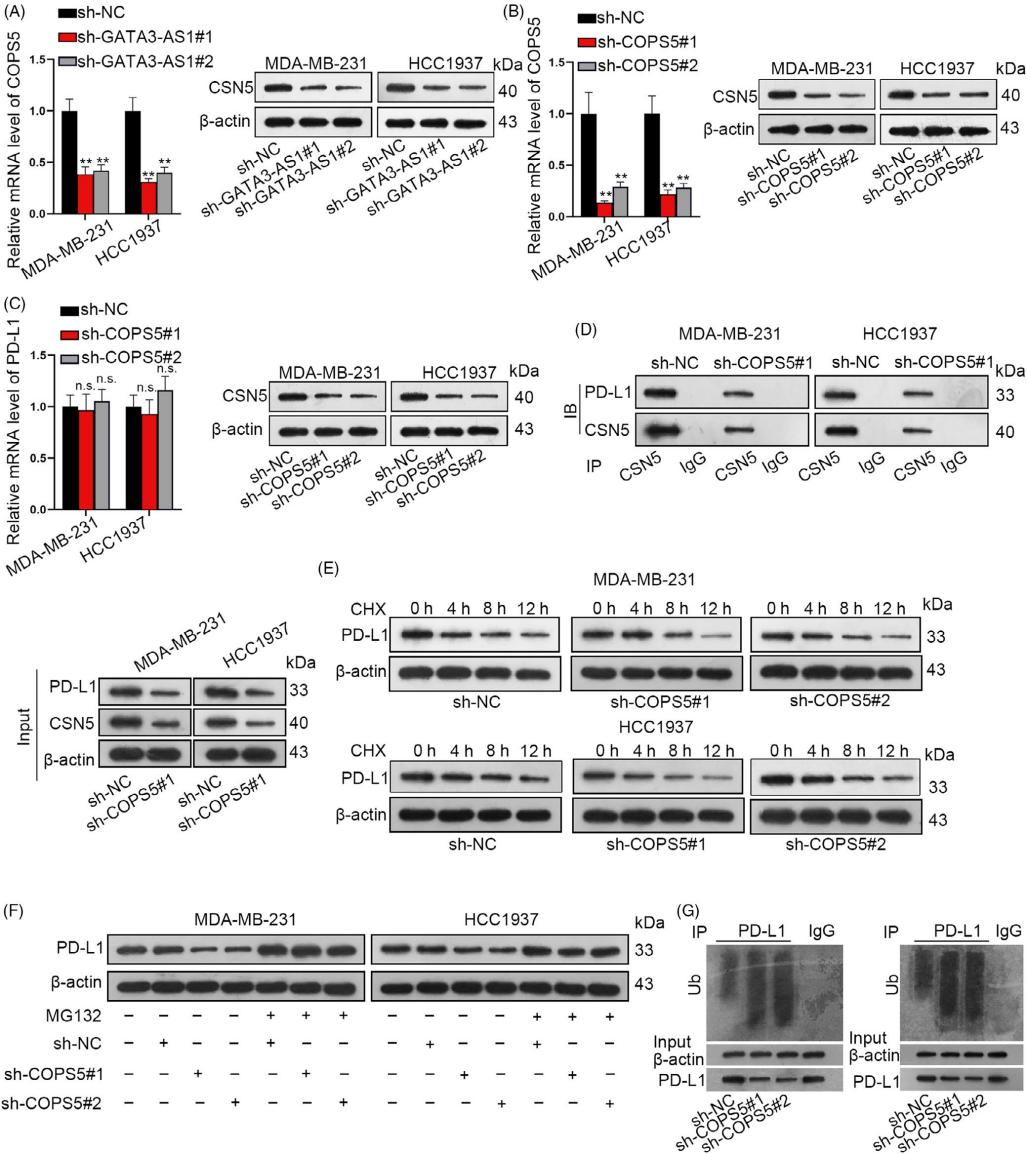
GATA3‐AS1 positively regulated COPS5/CSN5 to reverse the deubiquitination of PD‐L1. A, The potential regulation of GATA3‐AS1 on the mRNA or protein level of CSN5 was observed in TNBC cells transfected with sh‐GATA3‐AS1 or sh‐NC. B‐C, The effect of COPS5 silence on the level of PD‐L1 was investigated through qRT‐PCR and Western blot analyses. D, The interaction between endogenous PD‐L1 and CSN5 was further demonstrated by co‐IP assay followed by Western blot analysis. E, The half‐life of PD‐L1 protein was assessed in TNBC cells treated with CHX after silencing of COPS5. F, Protein level of PD‐L1 in MG‐132–treated TNBC cells was examined after COPS5 was silenced. G, The potential effect of COPS5 knockdown on the ubiquitination level of PD‐L1 was measured by ubiquitination assay. **P < .01. n.s.: not significant

Previously, PD‐L1 was uncovered to be stabilized by its deubiquitination regulator CSN5.^9^ In current study, we discovered that CSN5 expression was decreased by GATA3‐AS1 deficiency (Figure 3A). After silencing COPS5 in MDA‐MB‐231 and HCC1937 cells (Figure 3B), PD‐L1 was downregulated by silenced COPS5 at protein level but not mRNA level (Figure 3C). The interaction between endogenous PD‐L1 and CSN5 was further demonstrated by Co‐IP assay followed by Western blot analysis (Figure 3D). Afterwards, we found that the half‐life of PD‐L1 protein was decreased by COPS5 depletion (Figure 3E). Additionally, in MG‐132–treated TNBC cells, no significant changes were observed after COPS5 was silenced (Figure 3F). Further, silenced COPS5 promoted PD‐L1 ubiquitination (Figure 3G). Briefly, GATA3‐AS1 promoted PD‐L1 deubiquitination by upregulating COPS5.

### 3.4 GATA3‐AS1 induced COPS5 upregulation by sequestering miR‐676‐5p

**FIGURE 4:**
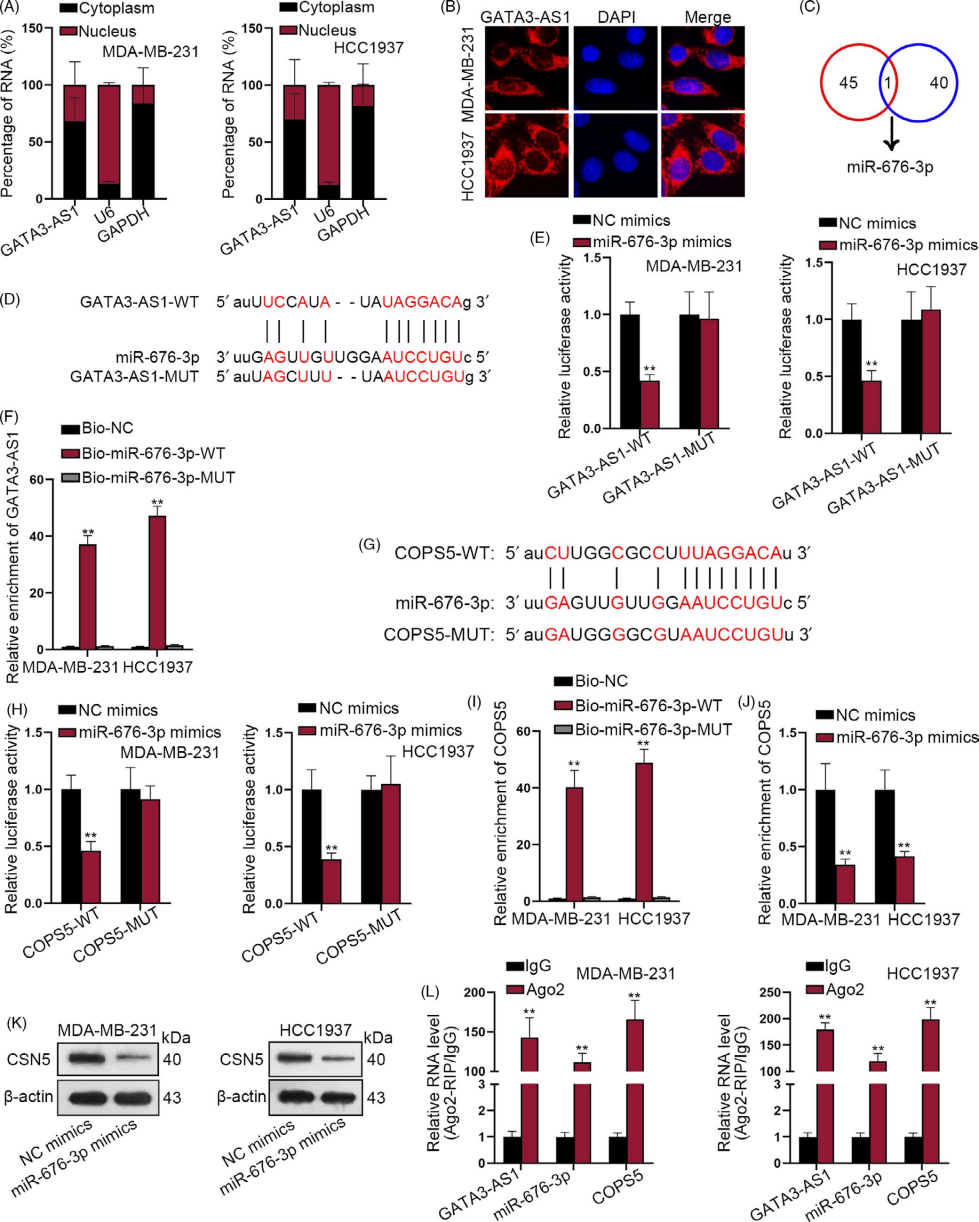
GATA3‐AS1 induced COPS5 upregulation by sequestering miR‐676‐5p. A‐B, The cellular localization of GATA3‐AS1 in TNBC cells was identified by subcellular fractionation and FISH assay. C, miRNA that can bind with both GATA3‐AS1 and COPS5 was searched out by bioinformatic analysis. D, The binding sequences between miR‐676‐3p and GATA3‐AS1 were predicted and illustrated. E, Luciferase reporter assays were conducted to demonstrate the interaction between GATA3‐AS1 and miR‐676‐3p. F, RNA pull‐down assay further demonstrated the enrichment of GATA3‐AS1 in complex pulled down by biotinylated wild‐type miR‐676‐3p, bio‐NC or bio‐miR‐676‐3p‐MUT. G, The binding sequence between miR‐676‐3p and COPS5 was also obtained and shown. H‐I, Luciferase reporter assay and biotinylated RNA pull‐down assay were used to determine the interaction between miR‐676‐3p and COPS5 in two TNBC cells. J‐K, The expression level of COPS5 was assessed in response to the upregulation of miR‐676‐3p in two TNBC cells. L, Ago2‐RIP assay was utilized to prove the enrichment of GATA3‐AS1, miR‐676‐3p and COPS5 were enriched in RISC complex. **P < .01

Since GATA3‐AS1 was predominantly located in cytoplasm of TNBC cells (Figure 4A,B), we further predicted the miRNAs that potentially bind with GATA3‐AS1. Here, we predicted that miR‐676‐5p could bind with GATA3‐AS1 and COPS5 through bioinformatic analysis (Figure 4C). The binding sequence between miR‐676‐3p and GATA3‐AS1 was predicted and illustrated in Figure 4D. Then, luciferase reporter assay demonstrated that the luciferase activity of GATA3‐AS1‐WT was decreased by miR‐676‐3p upregulation (Figure 4E). RNA pull‐down assay further demonstrated that GATA3‐AS1 was significantly pulled down by Bio‐miR‐676‐3p‐WT (Figure 4F). Subsequently, the binding sequence between miR‐676‐3p and COPS5 was also obtained and shown in Figure 4G. Moreover, luciferase reporter RNA pull‐down assays determined the interaction between miR‐676‐3p and COPS5 (Figure 4H,I). Besides, miR‐676‐3p upregulation decreased COPS5 expression (Figure 4J,K). Finally, Ago2‐RIP assay proved that GATA3‐AS1, miR‐676‐3p and COPS5 were enriched in RISC complex (Figure 4L), further indicating the ceRNA pathway. Additionally, COPS5 expression was upregulated in TNBC cells (Figure S2A,B). Further, the role of miR‐676‐3p and COPS5 in regulating the percentage and apoptosis rate of CD8^+^ T cells was also detected. As shown in Figure S2C,D, miR‐676‐3p upregulation increased the percentage of CD8^+^ T cells and decreased the apoptosis rate of CD8^+^ T cells, which presented the same effect with silenced COPS5 (Figure S2E,F).

### 3.5 GATA3‐AS1 induced the degradation of GATA3 protein

**FIGURE 5:**
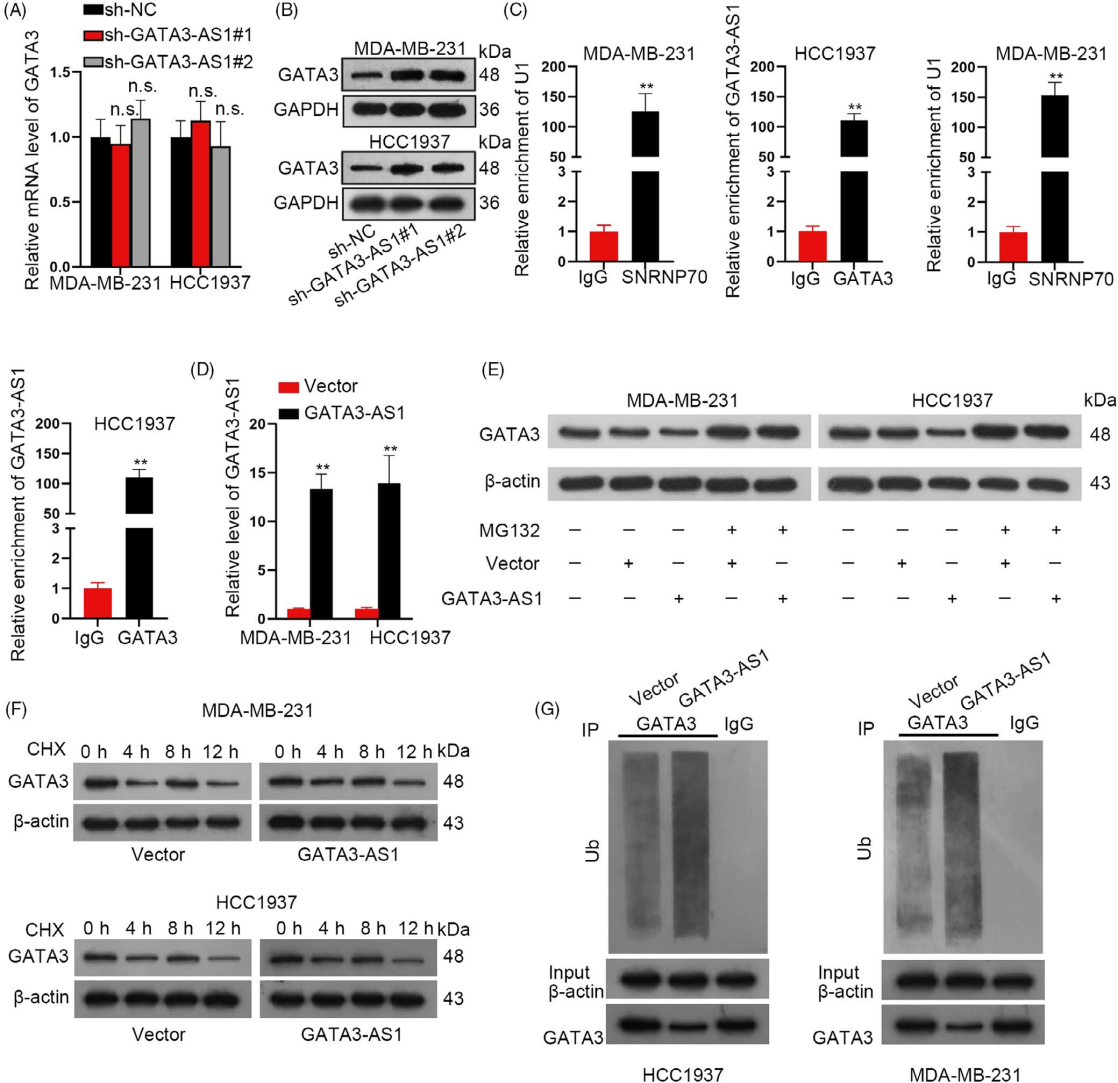
GATA3‐AS1 induced the degradation of GATA3 protein. A‐B, The expression of GATA3 mRNA and protein was assessed after downregulation of GATA3‐AS1 in TNBC cells. C, RIP assay demonstrated the direct interaction between GATA3‐AS1 and GATA3 protein in TNBC cells. D, Overexpressed GATA3‐AS1 in two TNBC cells was identified by qRT‐PCR analysis. E‐F, Western blot assay in cells treated with CHX or MG132 was conducted to determine the protein level of GATA3 in response to GATA3‐AS1 overexpression. G, The ubiquitination assay was conducted in response to the upregulation of GATA3‐AS1. **P < .01. n.s.: not significant

Previous reports have revealed that lncRNAs can exert functions by regulating their nearby genes.^15^, ^16^, ^17^ Here, we wondered whether GATA3‐AS1 regulated GATA3 in TNBC. As shown in Figure 5A,B, GATA3‐AS1 depletion elevated GATA3 protein level but not mRNA level (Figure 5A,B). RIP assay demonstrated that GATA3‐AS1 bound with GATA3 (Figure 5C). After overexpressing GATA3‐AS1 (Figure 5D), GATA3 protein level increased by MG132 was not affected (Figure 5E). However, in CHX‐treated TNBC cells, we observed that the half‐life of GATA3 protein was decreased efficiently by GATA3‐AS1 overexpression (Figure 5F). Interestingly, upregulation of GATA3‐AS1 induced GATA3 ubiquitination (Figure 5G). In this regard, we confirmed that high level of GATA3‐AS1 promoted the ubiquitination of GATA3 protein. To demonstrate the role of GATA3 in GATA3‐AS1–mediated TNBC progress, we also performed rescue assays. After GATA3 was silenced in TNBC cells (Figure S3A), the suppressive effect of GATA3‐AS1 knockdown on cell proliferation and migration was reversed (Figure S3B‐D).

### 3.6 High level of GATA3‐AS1 in TNBC cells was attributed to CBP‐mediated H3K27 acetylation

**FIGURE 6:**
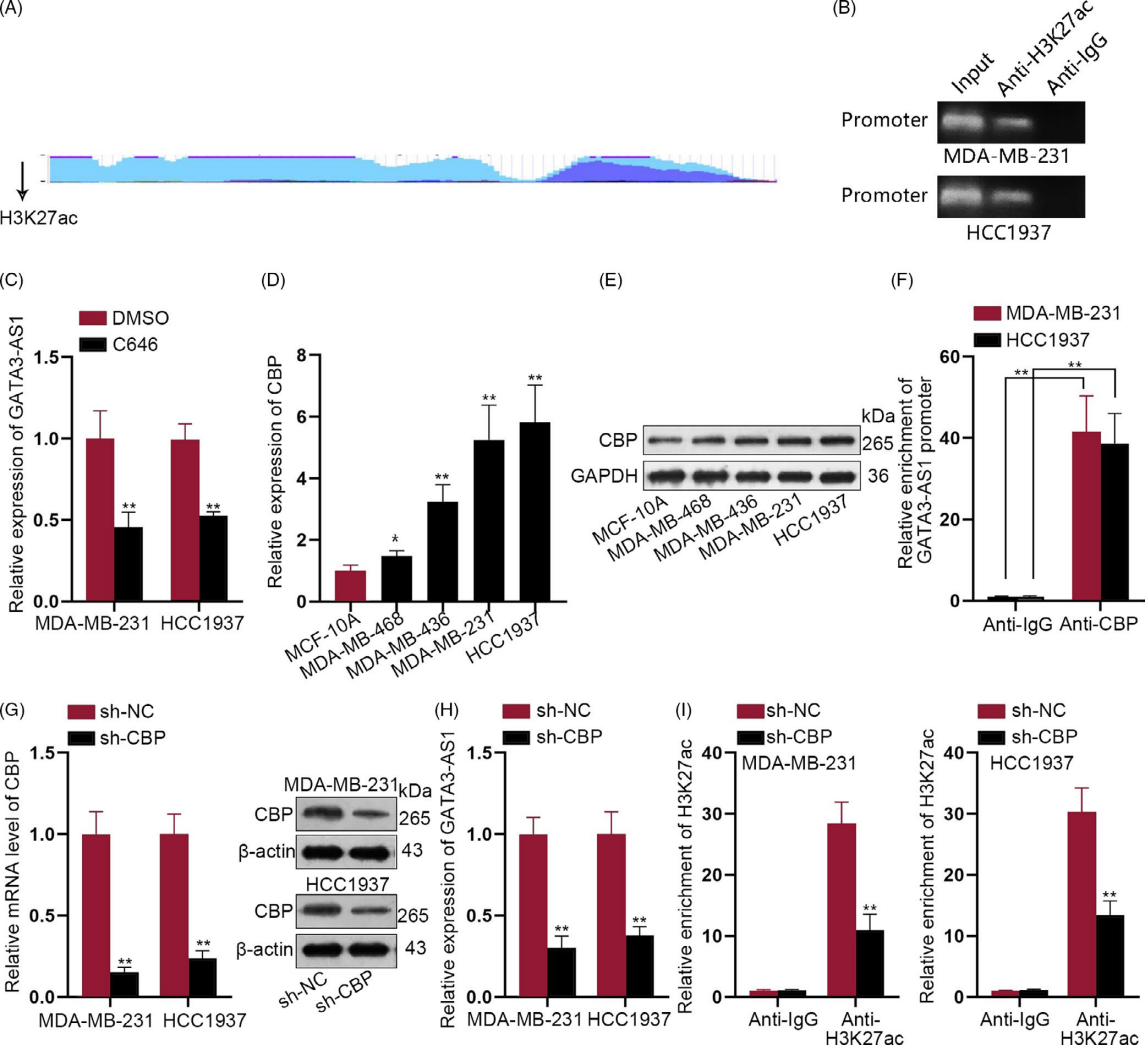
High level of GATA3‐AS1 in TNBC cells was attributed to CBP‐mediated H3K27 acetylation. A, High density of H3K27ac enrichment in GATA3‐AS1 promoter was uncovered in online database UCSC. B, ChIP assay was carried out with the anti‐H3K27ac antibody to demonstrate the histone acetylation in GATA3‐AS1 promoter. C, The corresponding level of GATA3‐AS1 was measured in TNBC cells treated with or without the histone acetyltransferase (HAT) inhibitor C646. D‐E, The expression level of CBP in TNBC and normal cells was analysed by qRT‐PCR assay and Western blot assay, respectively. F, ChIP assay was applied to determine the enrichment of CBP in GATA3‐AS1 promoter region. G, CBP was silenced in two TNBC cells and was confirmed by qRT‐PCR analysis and Western blot. H, GATA3‐AS1 expression was observed in CBP‐downregulated TNBC cells. I, ChIP assay demonstrated the effect of silenced CBP on the enrichment of H3K27ac in GATA3‐AS1 promoter. *P < .05, **P < .01

Then, we screened UCSC database and found the promoter region of GATA3‐AS1 with high density of H3K27ac enrichment (Figure 6A). To demonstrate the histone acetylation in GATA3‐AS1 promoter, ChIP assay was carried out. As illustrated in Figure 6B, H3K27ac was enriched in GATA3‐AS1 promoter. Furthermore, we observed a significant downregulation of GATA3‐AS1 in response to the treatment of C646, the histone acetyltransferase (HAT) inhibitor (Figure 6C). In addition, we detected CBP expression in TNBC and normal cells. Relative higher level of CBP was observed in four TNBC cells, especially in MDA‐MB‐231 and HCC1937 cells (Figure 6D,E). Through ChIP assay, we determined that CBP was enriched in GATA3‐AS1 promoter region (Figure 6F). After silencing CBP in two TNBC cells (Figure 6G), GATA3‐AS1 expression was downregulated (Figure 6H). Finally, ChIP assay demonstrated that silenced CBP led to the decreased enrichment of H3K27ac in GATA3‐AS1 promoter (Figure 6I). Collectively, CBP‐mediated H3K27ac enrichment in GATA3‐AS1 promoter induced GATA3‐AS1 upregulation in TNBC cells.

### 3.7 GATA3‐AS1 promoted TNBC cell growth and metastasis in vivo and predicted poor prognosis in TNBC patients

**FIGURE 7:**
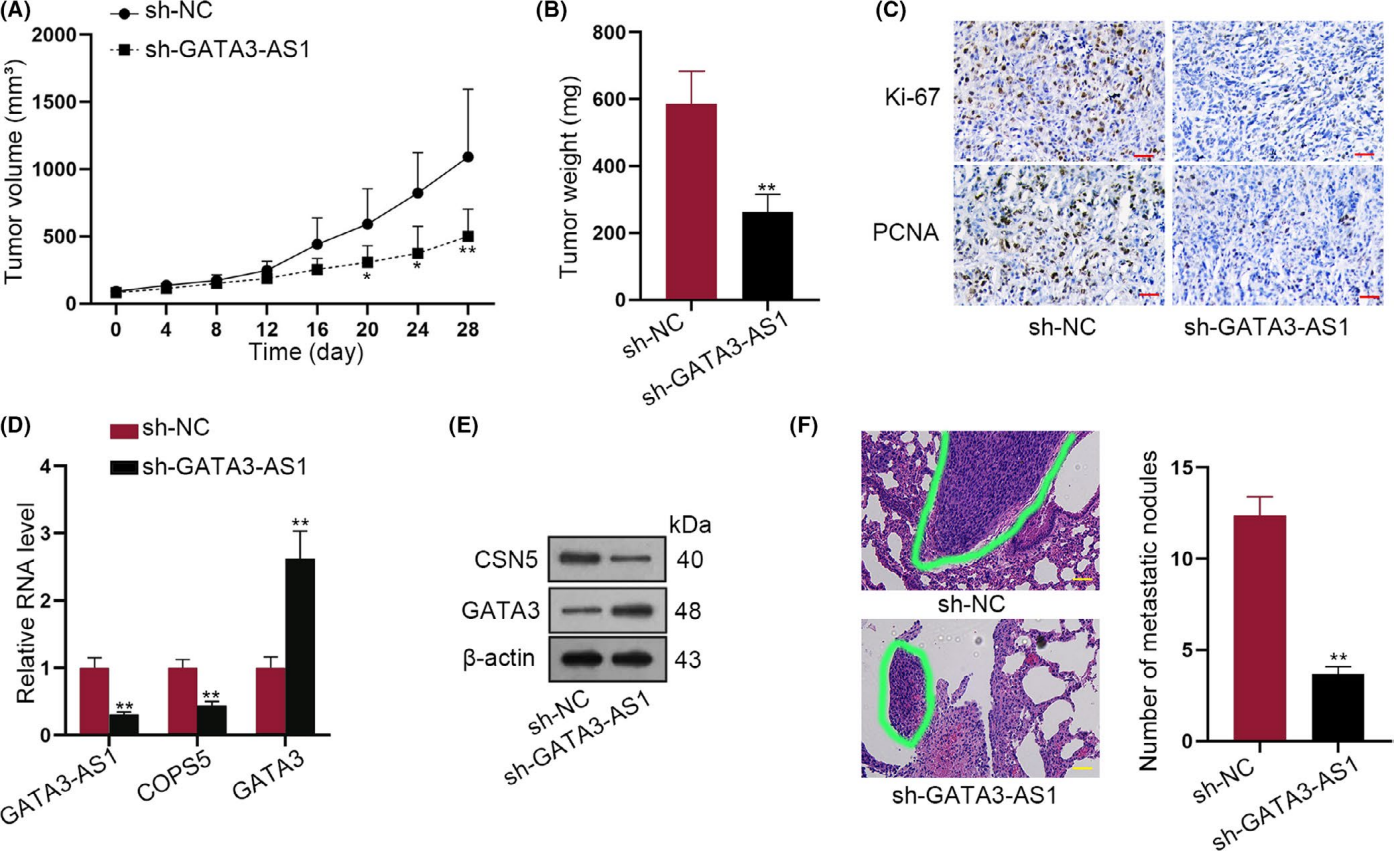
GATA3‐AS1 promoted TNBC cell growth and metastasis in vivo. A‐B, Stably transfected MDA‐MB‐231 cells with sh‐NC or sh‐GATA3‐AS1 were inoculated into the nude mice (five in each groups). Tumours were resected 28 d after inoculation. Tumour size and tumour weight were carefully counted and compared. C, The positivity of Ki‐67 and PCNA was also observed in tumour tissues in sh‐GATA3‐AS1 group or sh‐NC group. D, The expression level of GATA3‐AS1, COPS5, PD‐L1 and GATA3 in two groups was detected by qRT‐PCR analysis. E, The protein levels of CSN5. PD‐L1 and GATA3 were examined in two different groups. F, The metastatic nodule was calculated in sh‐GATA3‐AS1 group and sh‐NC group by in vivo metastasis assay. *P < .05, **P < .01

**FIGURE 8:**
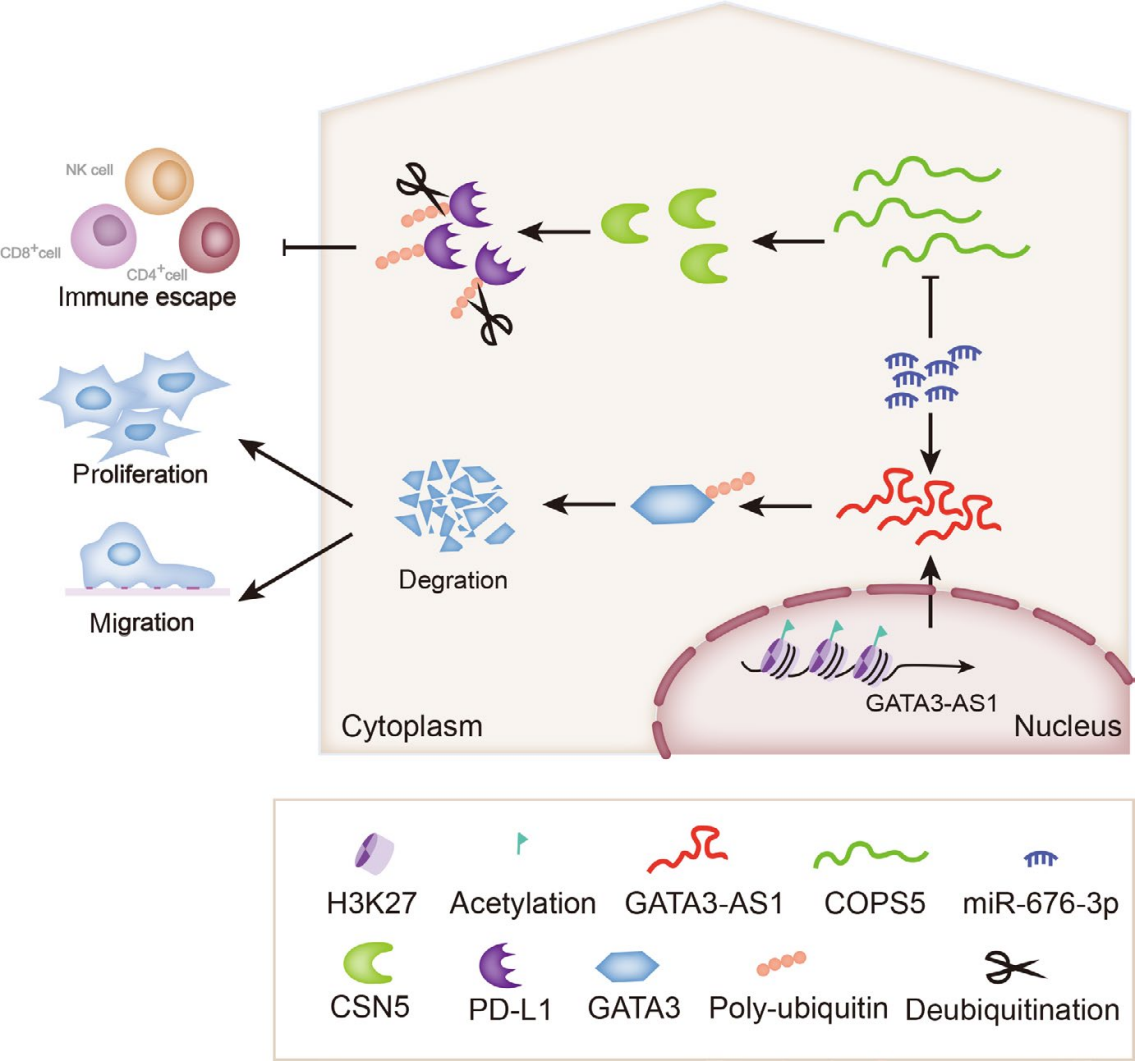
Illustration of mechanism by which GATA3‐AS1 functions in TNBC

Finally, in vivo experiments were conducted to prove the effect of GATA3‐AS1 on TNBC tumour growth and metastasis. As illustrated in Figure 7A,B, tumour growth was slower in GATA3‐AS1–downregulated group, which was reflected in tumour volume and tumour weight (Figure 7A,B). The lower positivity of Ki‐67 and PCNA was also observed in sh‐GATA3‐AS1 group (Figure 7C). Then, qRT‐PCR analysis revealed that the expression of GATA3‐AS1 and COPS5 was increased in sh‐GATA3‐AS1 group, which was opposite with that of GATA3 (Figure 7D). Moreover, GATA3‐AS1 downregulation decreased CSN5 protein level whereas elevated GATA3 protein level (Figure 7E). In vivo metastasis assay also demonstrated that the metastatic nodule in sh‐GATA3‐AS1 group was less than sh‐NC group (Figure 7F). Clinical significance of GATA3‐AS1/PD‐L1 was also detected. GATA3‐AS1 was highly expressed in TNBC tissues (Figure S4A). And clinical data uncovered that high level of GATA3‐AS1 was correlated large tumour size, lymph node metastasis and high TNM stage (Table S1). Kaplan‐Meier analysis revealed that patients with high GATA3‐AS1 level had lower overall survival rate (Figure S4B). Consistently, we observed the high level of PD‐L1 in TNBC tissues and PD‐L1 upregulation predicted poor prognosis (Figure S4C,D). High level of PD‐L1 was correlated with large tumour size and high TNM stage (Table S2). The expression correlation between GATA3‐AS1 and PD‐L1 in TNBC tissues was found to be positive (Figure S4E). Finally, ISH showed that GATA3‐AS1 and PD‐L1 were highly expressed in TNBC patient samples (Figure S4F). Combining with previous findings, we concluded that GATA3‐AS1 promoted TNBC proliferation and migration both in vitro and in vivo (Figure 8).

## 4 DISCUSSION

LncRNAs can involve in multiple events in human cancers, including tumour growth, metastasis, angiogenesis, drug resistance, tumour microenvironment regulation and self‐renewal of cancer cells.^18^, ^19^ Recent studies have revealed the vital functions of various abnormally expressed lncRNAs in TNBC. For example, Augoff K et al reported a lncRNA LOC554202 is regulated by promoter hypermethylation in TNBC.^20^ LncKLHDC7B is enriched in TNBC and contributes to the migration and resistance to death of TNBC cells.^21^ LncRNA MIR100HG with triplex formation with p27 loci and exerts oncogenic property in TNBC.^22^ In our current study, we observed from online databases that GATA3‐AS1 was overexpressed in BC patient samples, thus GATA3‐AS1 was chosen as our research object. Previously, lncRNA GATA3‐AS1 has been reported as a potential participant in T helper 2 Cells ^23^ and expressed in CD4^+^ T cell.^24^ Functionally, silenced GATA3‐AS1 suppressed TNBC cell proliferation and migration, but induced cell apoptosis. Thus, we supposed that GATA3‐AS1 might act as a potential tumour‐promoter in TNBC.

Immune escape of cancer cells to CD8^+^ T cells is a very important inducement for the cancer progression. The regulatory effect of GATA3‐AS1 on T cells was also revealed as previously reported. LncRNAs have been reported to be regulators that can affect its ubiquitination, thus promoting or suppressing its degradation.^25^, ^26^, ^27^, ^28^ Based on this point, we detected that GATA3‐AS1 promoted the degradation of PD‐L1 by affecting its ubiquitination level. A previous literature recorded that PD‐L1 protein could be stabilized by CSN5.^9^ In our present study, we found that CSN5 was positively regulated by GATA3‐AS1 at both mRNA and protein level. It is known that cytoplasmic lncRNAs can act as positive regulators for mRNAs by sequestering miRNAs.^29^, ^30^, ^31^, ^32^ Combing with all these findings, we confirmed that GATA3‐AS1 promoted the immune escape of TNBC cells by regulating CSN5‐mediated deubiquitination of PD‐L1.

LncRNAs have been reported to be able to regulate their nearby genes, thus affecting tumour progression. In our study, we uncovered that GATA3‐AS1 regulated its nearby gene GATA3 at protein level, GATA3, a previously reported tumour suppressor in human cancers.^33^, ^34^ In current study, we also examined whether GATA3‐AS1 mediated the degradation of GATA3 protein.

In our current study, GATA3‐AS1 and PD‐L1 were upregulated in TNBC tissues and correlated with the poor prognosis of TNBC patients. Moreover, GATA3‐AS1 and PD‐L1 had a positive correlation in TNBC patient samples. PD‐1/PD‐L1 immune checkpoint blockade has been studied due to its value in treating BC.^35^, ^36^ This study explored the upstream molecular mechanism of PD‐L1 in TNBC, which might contribute to unveil the novel and possible strategy for the immunotherapy of TNBC. Additionally, GATA3‐AS1 might be a therapeutic target in TNBC.

## CONFLICT OF INTEREST

None.

## AUTHORS' CONTRIBUTIONS

Ming Zhang conceived and designed the research; Ming Zhang and Ning Wang realized, analysed and interpreted the data; Peng Song and Yingqiang Fu wrote the paper including the design of figures; Ming Zhang and Yanlv Ren prepared materials; Zhigao Li and Jinsong Wang critically revised the manuscript for important intellectual content. All authors read and approved the final manuscript.

## Supporting information

Fig S1

Fig S2

Fig S3

Fig S4

Table S1

Table S2

Supplementary Material

## Data Availability

Research data are not shared.
