# Supplementary figures and images for: LncRNA GATA3‐AS1 facilitates tumour progression and immune escape in triple‐negative breast cancer through destabilization of GATA3 but stabilization of PD‐L1
Source: Cell Prolif. 2020 Jul 20;53(9):e12855. doi: 10.1111/cpr.12855 (PMC7507373; doi:10.1111/cpr.12855)

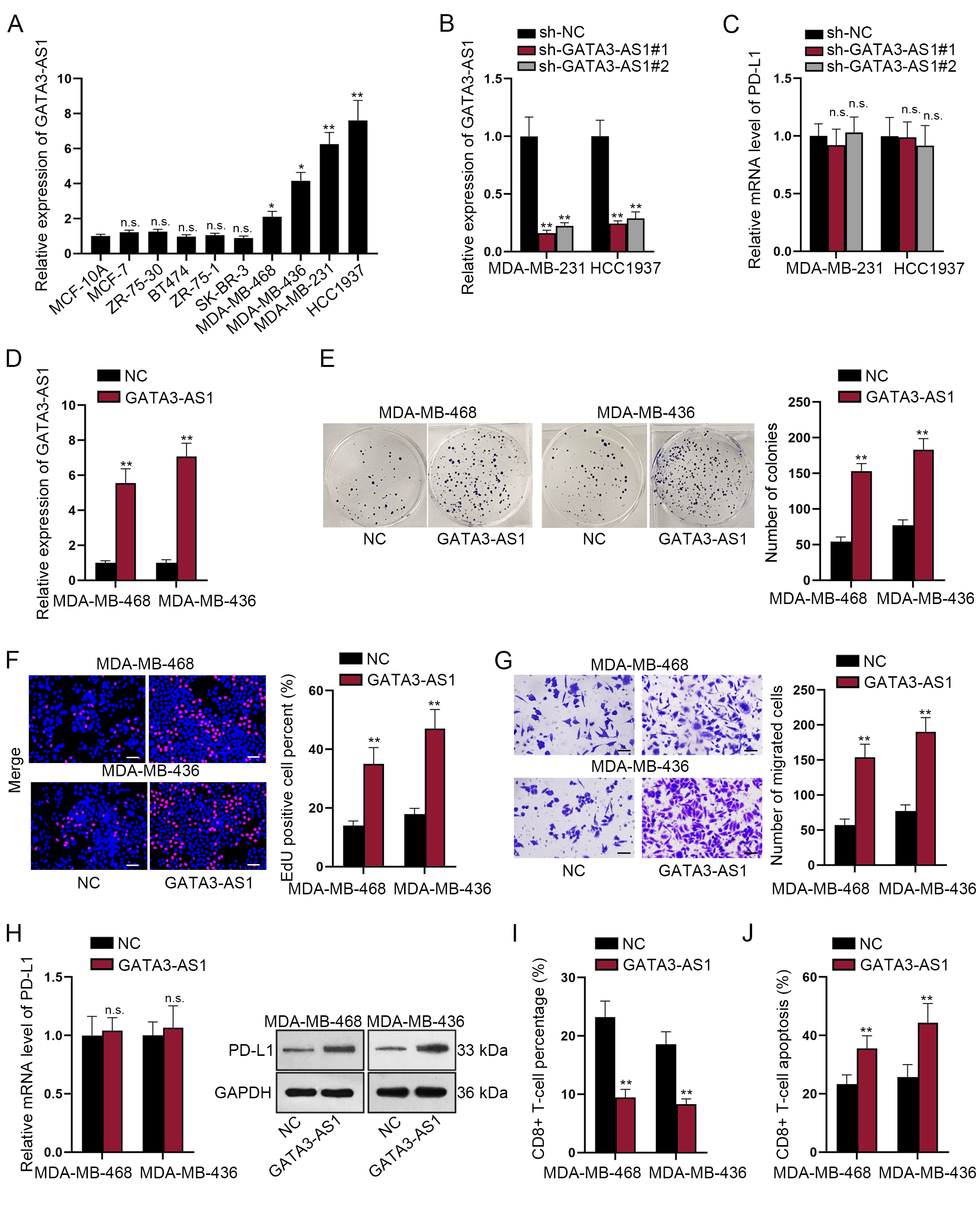

Supplement: Supplementary file 1 — Fig S1 [file CPR-53-e12855-s001.tif]

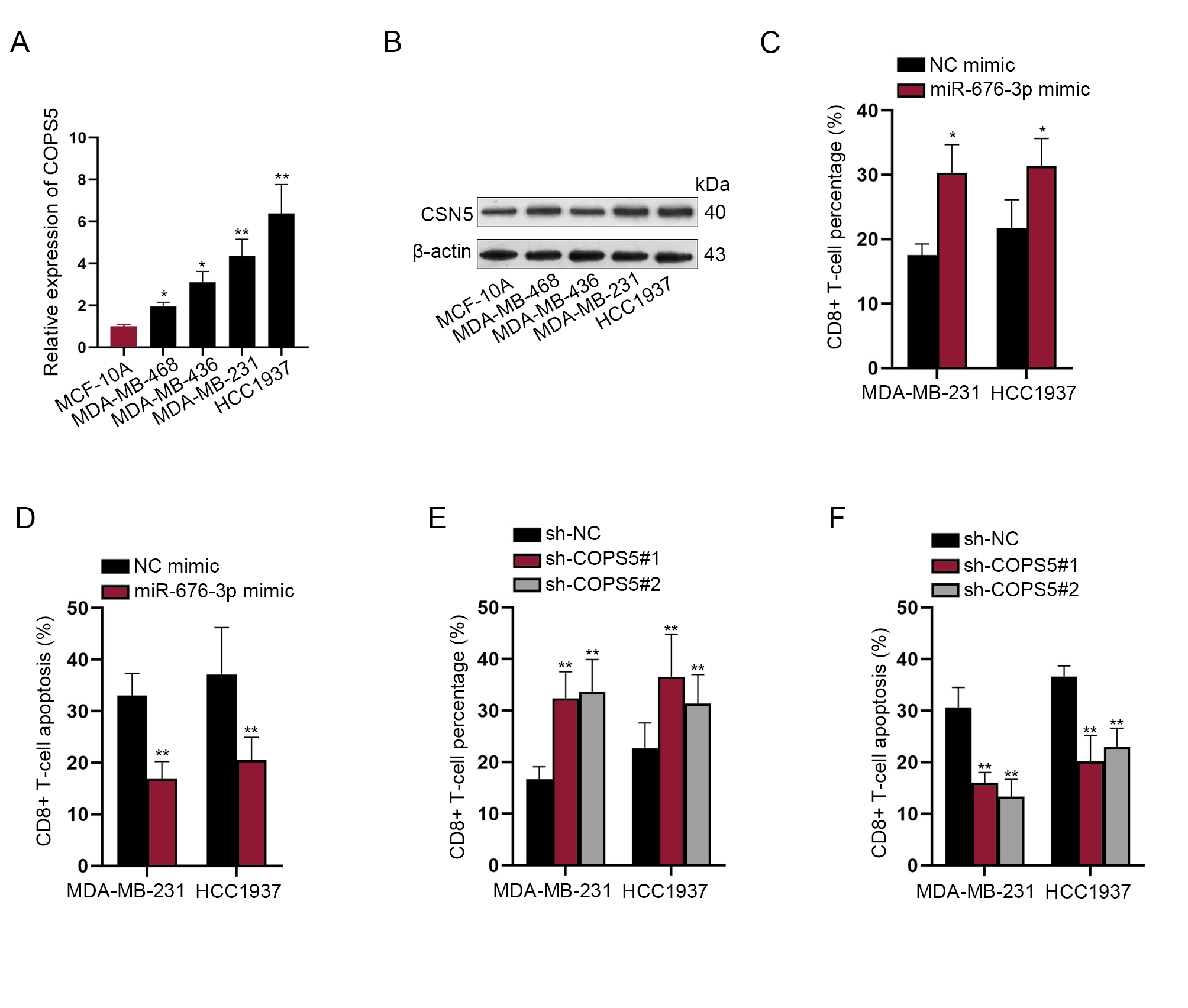

Supplement: Supplementary file 2 — Fig S2 [file CPR-53-e12855-s002.tif]

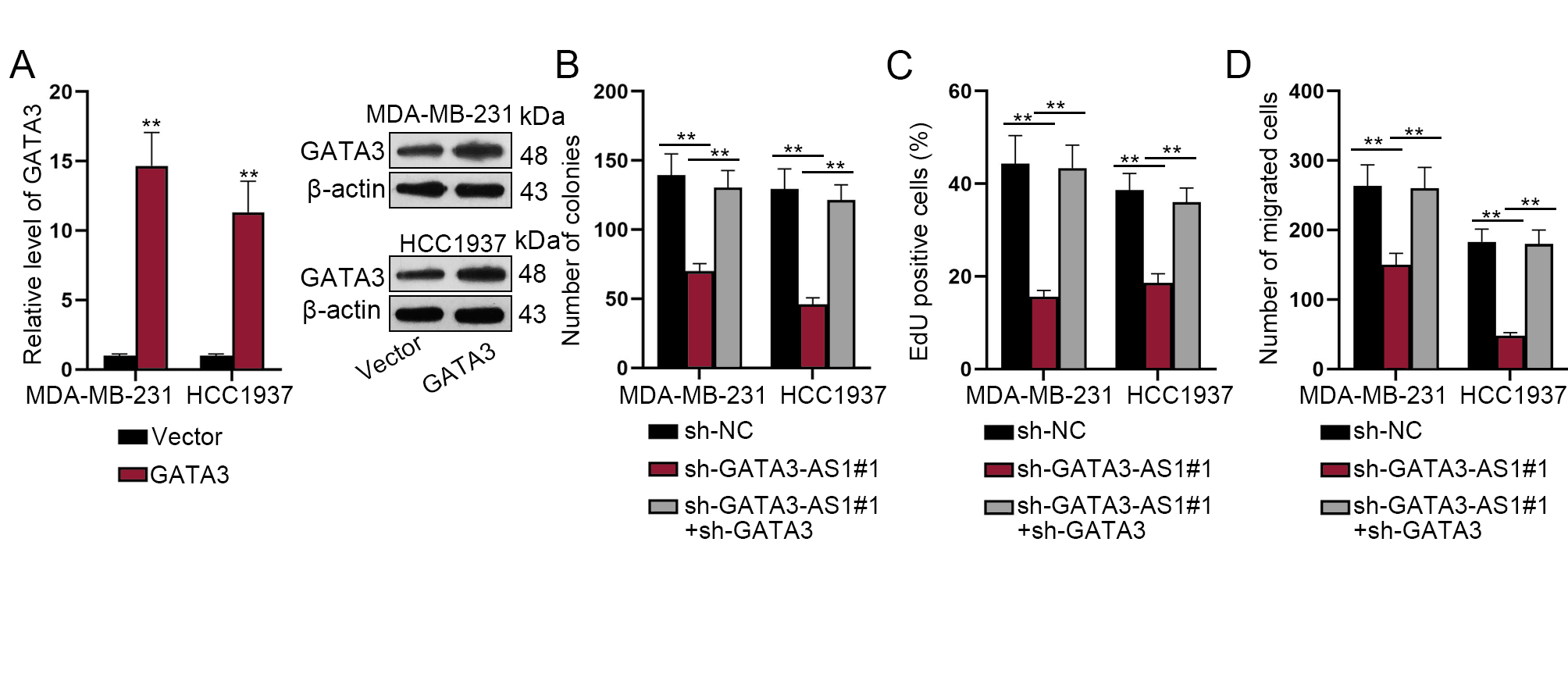

Supplement: Supplementary file 3 — Fig S3 [file CPR-53-e12855-s003.tif]

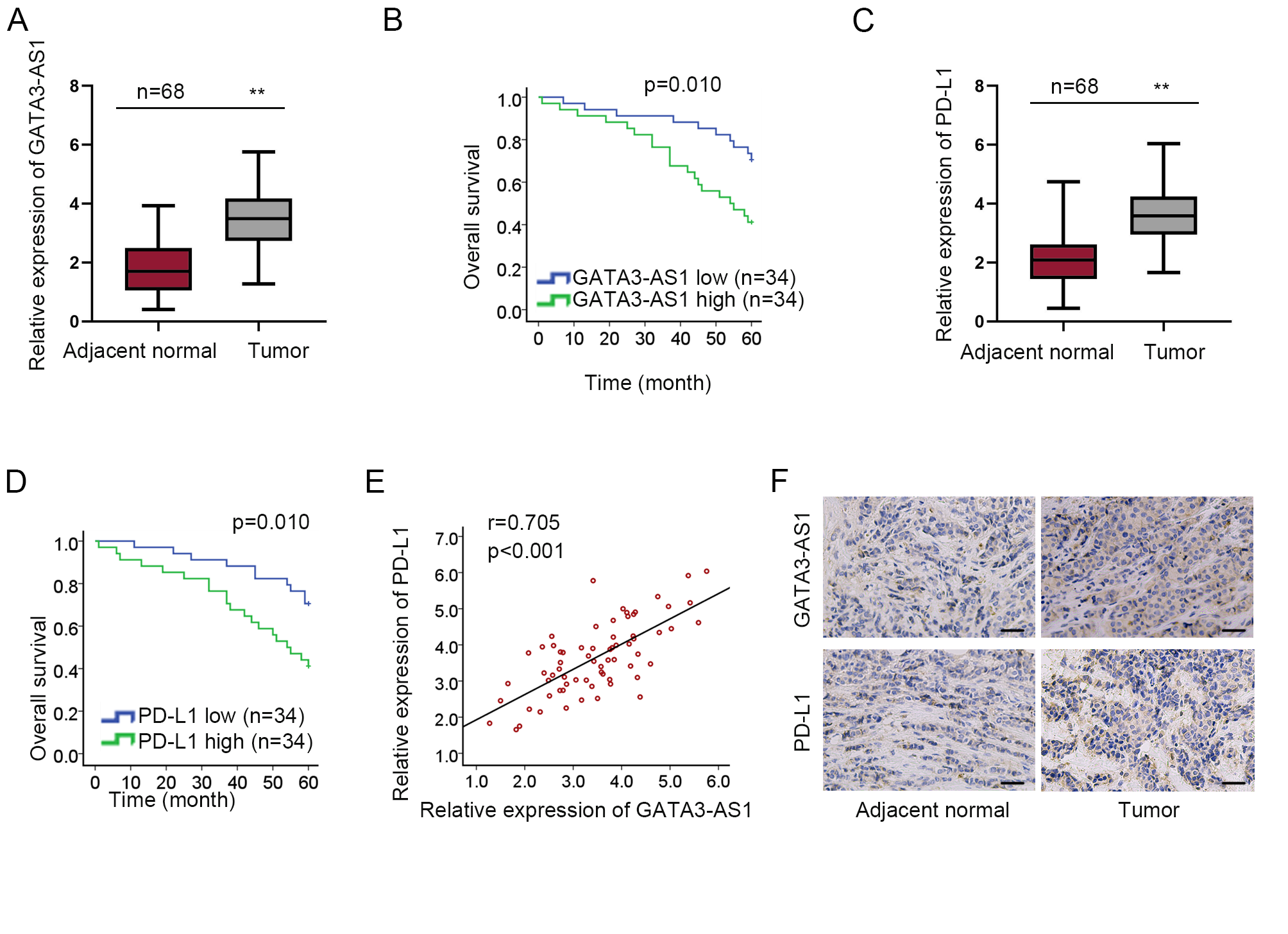

Supplement: Supplementary file 4 — Fig S4 [file CPR-53-e12855-s004.tif]
