# Supplementary material for: LncRNA GATA3‐AS1 facilitates tumour progression and immune escape in triple‐negative breast cancer through destabilization of GATA3 but stabilization of PD‐L1
Source: Cell Prolif. 2020 Jul 20;53(9):e12855. doi: 10.1111/cpr.12855 (PMC7507373; doi:10.1111/cpr.12855)
Supplement: Supplementary file 6 — Table S2 [file CPR-53-e12855-s006.docx]

**Supplementary Table 2.** Correlation between PD-L1 Expression and Clinical Features of TNBC patients. (n=68)

| Variable | PD-L1 Expression | | P-value |
| --- | --- | --- | --- |
|  | low | high |  |
| **Age** |  |  |  |
| <50 | 23 | 22 | 1.000 |
| ≥50 | 11 | 12 |  |
| **Menopause** |  |  |  |
| No | 31 | 25 | 0.190 |
| Yes | 3 | 9 |  |
| **Tumor size** |  |  |  |
| <2 cm | 22 | 11 | 0.015^*^ |
| ≥2 cm | 12 | 23 |  |
| **Lymph node metastasis** |  |  |  |
| No | 16 | 13 | 0.624 |
| Yes | 18 | 21 |  |
| **TNM** |  |  |  |
| I-II | 20 | 5 | <0.001^***^ |
| III-IV | 14 | 29 |  |
|  |  |  |  |

Low/high by the sample median. Pearson χ^2^ test. ^*^P<0.05, ^***^P<0.001 was considered to be statistically significant.
