# Supplementary material for: LncRNA GATA3‐AS1 facilitates tumour progression and immune escape in triple‐negative breast cancer through destabilization of GATA3 but stabilization of PD‐L1
Source: Cell Prolif. 2020 Jul 20;53(9):e12855. doi: 10.1111/cpr.12855 (PMC7507373; doi:10.1111/cpr.12855)
Supplement: Supplementary file 7 — Supplementary Material [file CPR-53-e12855-s007.docx]

**Figure S1 Overexpression of GATA3-AS1 promoted TNBC cell growth and migration.** (**A**) GATA3-AS1 level in MCF-10A cell and different subtypes of BC cells. (**B**) Silence of GATA3-AS1 by specific two shRNAs were confirmed by qRT-PCR, and non-targeting shRNA was used as negative control (sh-NC). (**C**) The mRNA level of PD-L1 was assessed in TNBC cells transfected with GATA3-AS1–specific shRNAs compared to sh-NC–treated cells. (**D**) Overexpression efficiency of GATA3-AS1 in MDA-MB-468 and MDA-MB-436 cells. (**E-F**) Effect of GATA3-AS1 overexpression on the proliferation of two TNBC cells was assessed by colony formation assay and EdU assay. (**G**) Transwell migration assay was applied to evaluate the migratory ability of TNBC cells transfected with GATA3-AS1 expression vector. (**H**) The level of PD-L1 mRNA and protein was detected in cells transfected with GATA3-AS1 expression vector. (**I-J**) The percentage and the apoptosis rate of CD8^+^ T cells were evaluated in GATA3-AS1–upregulated TNBC cells. ^*^P < 0.05, ^**^P < 0.01. n.s.: not significant.

**Figure S2 The role of miR-676-5p and COPS5 on the percentage and apoptosis of CD8^+^ T cells.** (**A-B**) The mRNA and protein level of COPS5 was identified in TNBC cells compared to that in MCF-10A cell. ^*^P < 0.05, ^**^P < 0.01 indicated the statistical significance of data. (**C-D**) The percentage and the apoptosis rate of CD8^+^ T cells was detected in TNBC cells after upregulation of miR-676-3p. (**E-F**) The effect of COPS5 silencing on the percentage and the apoptosis rate of CD8^+^ T cells was detected in TNBC cells. ^*^P < 0.05, ^**^P < 0.01.

**Figure S3** (**A**) To demonstrate the role of GATA3 in GATA3-AS1–mediated TNBC progress, we also performed rescue assays. GATA3 was firstly overexpressed in TNBC cells for rescue assays. (**B-D**) Colony formation assay, EdU assay and transwell migration assay were separately conducted in cells transfected with sh-NC, sh-GATA3-AS1#1 or co-transfected with sh-GATA3-AS1#1+sh-GATA3. ^**^P < 0.01.

**Figure S4 Clinical significance of GATA3-AS1 and PD-L1 in TNBC.** (**A**) The level of GATA3-AS1 in paired TNBC tissues and adjacent normal tissues. (**B**) The overall survival rate of TNBC patients with high or low level of GATA3-AS1 was analyzed by Kaplan-Meier method. (**C**) PD-L1 level was assessed in TNBC tissues and adjacent normal tissues. (**D**) The correlation between PD-L1 expression and the overall survival rate of TNBC patients was assessed by Kaplan-Meier method. (**E**) Expression correlation between GATA3-AS1 and PD-L1 in TNBC tissues. (**F**) The expression level of GATA3-AS1 and PD-L1 was examined in TNBC patient tissues and adjacent tissues by In situ hybridization. ^**^P < 0.01.

**Supplementary Table 1 Correlation between GATA3-AS1 expression and clinical features of TNBC patients.** ^**^P < 0.01, ^***^P < 0.001.

**Supplementary Table 2 Correlation between PD-L1 expression and clinical features of TNBC patients.** ^*^P < 0.05, ^***^P < 0.001.
